# Dual imagery tasks for EEG-based brain–computer interfaces: a feasibility study of combinatorial mental state classification

**DOI:** 10.3389/fnhum.2026.1912998

**Published:** 2026-09-11

**Authors:** Hadi Mohammadpour, Sarah D. Power

**Affiliations:** 1 Department of Electrical and Computer Engineering, Faculty of Engineering and Applied Science, Memorial University of Newfoundland, St. John's, NL, Canada; 2 Division of Population Health and Applied Health Sciences, Faculty of Medicine, Memorial University of Newfoundland, St. John's, NL, Canada

**Keywords:** active brain–computer interface, dual mental imagery, electroencephalography, motor imagery (MI), multi-class brain–computer interface, singing imagery

## Abstract

**Introduction:**

Active brain–computer interfaces (BCIs) aim to enable communication and control without requiring physical movement. BCIs operate by detecting and decoding distinct patterns of brain activity associated with intentionally-generated mental tasks and translating them into commands for external devices. Motor imagery (MI) tasks involving the imagined movement of body parts are the most commonly used control paradigms in active BCIs; however, such systems are typically limited to a relatively small number of commands. Expanding the number of distinguishable mental states could increase the flexibility and information transfer capacity of active BCIs. In the present work dual imagery (DI) tasks are introduced. DI tasks involve the simultaneous performance of two mental tasks, specifically singing imagery (SI) combined with MI of one hand or both feet. This study investigated the feasibility of classifying neural activity associated with such combinatorial imagery tasks using EEG, thereby evaluating their potential to expand the number of distinguishable control states in active BCIs.

**Methods:**

EEG was recorded from 14 healthy participants while performing the specified mental tasks. Following pre-processing, a filter bank common spatial patterns (FBCSP) approach and a regularized linear discriminant analysis (LDA) classifier were used for feature extraction and classification in 3-, 4-, 7-, and 8-class scenarios. The 3-class analyses evaluated whether each DI task could be differentiated from its constituent single tasks, while the 4-class analyses incorporated a “rest” state. The 7- and 8-class scenarios explored the feasibility of increasing the number of distinguishable BCI control states beyond six.

**Results:**

Accuracies as high as 64.1% and 63.0% were achieved for the 3- and 4-class scenarios, respectively, demonstrating that DI tasks could be differentiated from both their constituent single tasks and a rest state. In the 7- and 8-class scenarios, average accuracies of approximately 55% and 50%, respectively, substantially exceeded the corresponding theoretical chance levels of 14.3% and 12.5%.

**Conclusion:**

While the accuracies obtained are currently insufficient for practical high-command active BCIs, the results demonstrate the feasibility of classifying neural activity associated with combinatorial imagery tasks. These findings support the potential of DI paradigms to expand the number of distinguishable control states in active BCIs.

## 1 Introduction

Brain–computer interfaces (BCIs) are an emerging technology that allows for direct communication between the brain and external devices. A BCI is defined as

“*A system that measures central nervous system (CNS) activity and converts it into artificial output that replaces, restores, enhances, supplements, or improves natural CNS output and thereby changes the ongoing interactions between the CNS and its external or internal environment”* (Wolpaw and Winter Wolpaw, 2012)

In particular, “active BCIs” provide users with a movement-free pathway to communicate and interact with their surroundings. The user controls the BCI by intentionally generating a predefined set of brain activity patterns which are detected and translated into commands for an external device.

Motor imagery (MI) tasks—typically, the imagined movement of the right hand (R), left hand (L), both feet (F), and tongue (T)—are the most commonly used tasks in EEG-based active BCI research (Blankertz, 2006; Tangermann et al., 2012). Less commonly, non-motor tasks such as mental calculation/counting (Myrden and Chau, 2015; Banville et al., 2017; Dutta et al., 2018; Sepulveda et al., 2007; Faradji et al., 2009; Friedrich et al., 2012; Scherer et al., 2013; Dobrea and Dobrea, 2009; Friedrich et al., 2013b,a; Obermaier et al., 2001), spatial navigation (Banville et al., 2017; Friedrich et al., 2012; Scherer et al., 2013; Friedrich et al., 2011, 2013b,a; Curran et al., 2004; Friedrich et al., 2013c), and singing imagery (Myrden and Chau, 2015; Banville et al., 2017) have also been considered in designing active BCIs. To date, the ability to differentiate as many as four or five different mental tasks at the same time, each of which would be associated with a command, has been investigated in active EEG-based BCI research (Blankertz, 2006; Tangermann et al., 2012; Christensen et al., 2019). Developing systems that support a higher number of commands is an important issue in this field of research (Yu et al., 2018) as it would significantly impact the practicality and user-friendliness of these systems by allowing the users to communicate at a higher information transfer rate and potentially giving access to a wider set of functions.

Our recent work (Mohammadpour and Power, 2025) thoroughly investigated the feasibility of using the mental task of singing imagery along with the four most common motor imagery tasks, both as an alternative to these tasks and as an option for increasing the number of system commands from five to six. Moreover, subjective data from participants suggested that singing imagery could be an intuitive, user-friendly control task. In particular, participants generally expressed their preference for the singing imagery task over the motor imagery of the tongue. Therefore, in the present work, we aimed to examine the potential of using singing imagery and motor imagery of limbs (i.e., right hand, left hand, and feet) to further increase the number of commands by developing a novel set of tasks, referred to as “dual imagery (DI)" tasks.

Dual imagery is a mental task involving the simultaneous performance of two usually distinct mental tasks. For instance, motor imagery of the left hand and the right hand, which are differentiable from one another by EEG, could be performed simultaneously to produce a third task. Although there is some disagreement as to the effects of multitasking on the brain (Adcock et al., 2000; Jaeggi et al., 2003), most agree that combining several low-level, simplified tasks has an additive effect (Hirshfield et al., 2011). Therefore, it is reasonable to suggest that combining two individual tasks would produce a third DI task with a distinct pattern of brain activity, such that all three tasks would be differentiable from one another. If so, using such DI tasks along with single tasks could be a novel approach to increasing the number of classes a BCI can detect and, in turn, the number of BCI commands. Detecting such dual tasks could be particularly suitable in applications like wheelchair/cursor control where the single tasks could be translated into movement in the four cardinal directions (i.e., north, east, south, and west), and their dual task could translate into movement in the corresponding ordinal direction (i.e., northeast, southeast, southwest, and northwest) (Kim et al., 2013).

Very few previous BCI studies have investigated DI tasks. One study of just three participants attempted to classify DI tasks in a 4-class scenario consisting of “no motor imagery,” “imagery of left hand,” “imagery of right hand,” and “imagery of both hands” (Geng et al., 2008). The study reported a maximum accuracy of 75.8% across the three participants. The potential of DI for increasing the number of commands beyond four was not investigated.

The feasibility of using DI tasks to increase the number of commands from four to five was explored in Reshmi and Amal (2013), where the simultaneous performance of R and L replaced the T imagery task. Moreover, Kim et al. (2013) also combined two motor imagery tasks to create a separate command; specifically, the simultaneous performance of R + F and L + F were used with the single tasks of R, L, and F to build a five-class system. Unfortunately, neither of these two studies reported performance evaluation metrics, so it is not possible to gauge the potential functionality of the proposed protocols.

Another study on DI tasks investigated the eight tasks that could be achieved by combining motor imagery of the left and right arms, and both feet (Takahashi and Tanaka, 2019). However, despite recording EEG data from 18 participants, they reported classification results for only those three that had distinguishable patterns (based on the BCI algorithm used in their study). Accuracies as high as 50% were reported for the 8-class classification across these three selected participants.

The previously investigated DI paradigms based on combining two MI tasks tend to be cognitively demanding and could lead to fatigue. Alternatively, the act of singing a song and tapping your hand or foot along to the rhythm is a very common experience for most people, so this could produce a relatively more intuitive and easier DI task combination. Furthermore, combining SI and MI tasks may generate more distinct composite neural patterns than combinations of similar MI tasks alone. Motivated by these considerations, in the present study, we investigate DI tasks created by the simultaneous performance of singing imagery with motor imagery of each hand and with the feet.

Here, based on the findings of our previous work, which investigated the potential of singing imagery as a BCI control task and verified the differentiability of SI and MI tasks (Mohammadpour and Power, 2025), the present study investigated DI tasks composed of a conventional motor imagery task performed simultaneously with singing imagery. Specifically, considering the MI tasks most commonly used in EEG-based BCI research (i.e., R, L, and F), each was combined with singing imagery to create a novel set of dual imagery tasks. In total, eight different tasks, including both single and dual imagery tasks, were considered (R, L, F, SI, R + SI, L + SI, F + SI, and REST). Limiting the investigation to this combination of tasks (and excluding DI combinations of two MI tasks, i.e., R + L, R + F, and L + F) enabled sufficient samples of each task to be recorded within a single experimental session. Task differentiability was then evaluated across multiple 3-, 4-, 7-, and 8-class classification scenarios to investigate whether simultaneous SI+MI tasks generate distinguishable composite neural states that could potentially expand the set of control states available for EEG-based active BCIs.

## 2 Methods

### 2.1 Participants

Fourteen healthy participants (mean age: 25.1 ± 11.6; seven male) were recruited for this study. All participants were right-handed. Because the study evaluated left- and right-hand motor imagery as separate control tasks, including participants with different dominant hands could have introduced additional variability into these comparisons. Participants were included if they had normal or corrected-to-normal vision and hearing, no cognitive impairment, and no history of neurological disease, disorder, or injury. Participants were asked to avoid exercising and consuming alcohol or caffeine for at least 4 h before the experiment. All participants provided written informed consent prior to completing the experiment. The experimental protocol was approved by the Interdisciplinary Committee for Ethics in Human Research (ICEHR) at Memorial University of Newfoundland.

Two participants withdrew after completing two experimental blocks because of tiredness and were therefore excluded from the analysis due to insufficient data.

### 2.2 Data acquisition

For this experiment, EEG data was recorded via a 64-channel actiCHamp system with active electrodes (Brain Products, GmbH), sampling at 500 Hz. The impedance for all electrodes was kept below 10 KΩ throughout the experimental session. Electrodes were placed according to the international 10–10 system. Markers indicating the timing of different mental tasks were transmitted via the TriggerBox (Brain Products, GmbH) to the recording software (Brain Vision Recorder).

### 2.3 Experimental protocol

**Figure 1:**
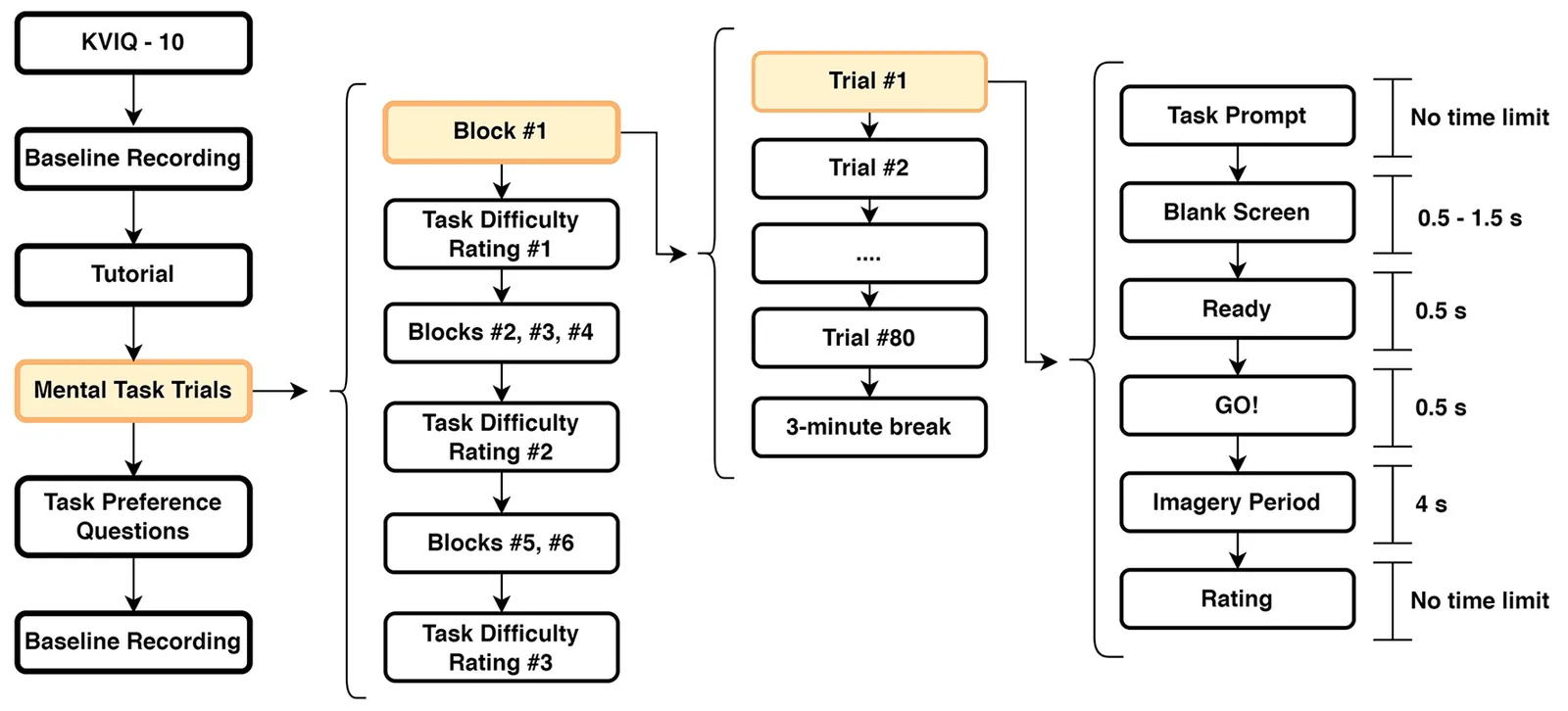
Experimental protocol illustrating the different steps of the single recording session.

Each participant completed a single experimental session during which they completed trials of each of the tasks of interest. The procedure of the experiment, which is summarized in Figure 1, is explained thoroughly in the following section.

The participants started by completing the Kinesthetic and Visual Imagery Questionnaire (KVIQ-10) (Malouin et al., 2007). This questionnaire is a standard set of tasks that are frequently used in BCI studies to subjectively assess the participants' ability to visualize and feel the sensations associated with kinesthetic imagined movements. The KVIQ-10 scores could potentially be helpful in interpreting the results of the MI trial data.

The participant was seated facing a computer screen for the duration of the experiment. The height of the chair and monitor were adjusted to the participant's comfort level. After the EEG electrodes were set up, two 1-min baseline trials, one with eyes open and the other with eyes closed, were recorded. These two baseline trials were repeated at the end of the experiment as well. Prior to beginning the motor imagery tasks, a brief tutorial explaining the experimental process and the imagery tasks was provided to the participant.

The participant then started performing the mental task trials. They were asked to perform eight different tasks, including rest. A detailed description of these tasks is given in Section 2.3.1 (i.e., “Mental Tasks"). Each task was performed 60 times for a total of 480 trials. Trials were completed in 6 blocks of 80 trials each (there were 10 trials per task per block). The task order was random in each block. The participants were instructed to take a break of at least 3 min between blocks.

The timeline for each trial is also shown in Figure 1. At the beginning of each trial, the task to be performed was displayed on the screen. Participants were able to initiate the timed portion of the trial, which lasted between 5.5 and 6.5 s, by pressing any key on the keyboard. The screen then turned blank for a brief period (0.5–1.5 s), followed by the messages “Ready!" (0.5 s) and then “GO!" (0.5 s). After this, a dark screen with only a “+" sign appeared, signaling the time for the participant to perform the mental task (4 s). The final step of each trial was the performance rating step.

The performance rating step, repeated at the end of each trial, was done in an attempt to capture the participant's perception of how well they performed the task. Specifically, they chose one of the following four options to indicate their feeling about their performance of the task: (1) Strong performance, (2) Moderate performance, (3) Weak performance, and (4) Wrong performance. Participants were asked to rate their performance based on factors such as the intensity of the kinesthetic sensations and their focus during the task imagination period. They were told to choose the option “(4) Wrong performance" if they either didn't perform the task, did the wrong task, or moved during the imagery period.

#### 2.3.1 Mental tasks

For this experiment, a singing imagery (SI) task, three single conventional motor imagery (MI) tasks, and three dual imagery (DI) tasks (i.e., the simultaneous performance of one of the MI tasks and SI) were included. A “rest" state was also included. These tasks were defined as follows:

- *Rest (REST)*: participants were told that they do not need to do anything specific for this task but were just asked not to perform any of the other seven tasks in the experiment. They were asked to keep their eyes open and focused on the screen throughout the trial (they could blink normally). No additional constraints were placed on participants' thoughts during the REST condition, as the intention was to represent a practical BCI scenario in which the user is not intentionally generating a control command.
- *Singing imagery (SI)*: participants imagined singing a song (with lyrics) in their heads, without vocalization or movement. Participants were instructed to focus on the kinesthetic sensation of moving their jaw, tongue, and lips as they imagined articulating the lyrics.
- *Left hand motor imagery (L)*: participants imagined tapping their left hand, bending at the wrist, at a steady pace of approximately one tap per second.
- Right hand motor imagery (R): participants imagined tapping their right hand, bending at the wrist, at a steady pace of approximately one tap per second.
- Feet motor imagery (F): participants imagined tapping both feet together, bending at the ankle, at a steady pace of approximately one tap per second.
- Dual left hand imagery (L_*SI*_): participants imagined performing both the left hand and singing imagery tasks simultaneously. Specifically, participants were instructed to imagine singing the lyrics of the song and tapping their left hand to the rhythm of the song.
- Dual right hand imagery (R_*SI*_): participants imagined performing both the right hand and singing imagery tasks simultaneously. Specifically, participants were instructed to imagine singing the lyrics of the song and tapping their right hand to the rhythm of the song.
- Dual feet imagery (F_*SI*_): participants imagined performing both the feet and singing imagery tasks simultaneously. Specifically, participants were instructed to imagine singing the lyrics of the song and tapping their feet to the rhythm of the song.

For the DI tasks, participants were instructed to focus on feeling the kinesthetic sensations of movements for both tasks (i.e., to imagine the feeling of moving their jaw, tongue, and lips while singing, and their hand or feet while tapping). For the SI and DI tasks, participants selected the songs to imagine from a list of widely known English songs (e.g., Jingle Bells, Happy Birthday To You, The Alphabet Song). A different song was chosen for each block of trials.

#### 2.3.2 Subjective evaluation of tasks

After completing blocks 1, 4, and 6, the participants were asked to rate the difficulty of each task. The rating was based on a scale of 1 to 5, where 1 represented “not difficult at all" and 5 represented “extremely difficult."

Additionally, after finishing all of the imagery trials, the participants were asked questions about their task preferences. They were asked the question:

“If you were to choose one of the tasks below to do on a daily basis to use a computer, which one would you pick?"

They were presented with the following three scenarios and asked to select one of the two options for each:

(1) **(a)** Single tasks, **(b)** dual tasks

(2) **(a)** Hand imagery + singing imagery, **(b)** feet imagery + singing imagery

(3) **(a)** Right hand + singing imagery, **(b)** left hand + singing imagery

### 2.4 EEG pre-processing

EEG data were pre-processed using the EEGLAB toolbox in MATLAB. Pre-processing included DC offset removal, average re-referencing, anti-alias filtering, down-sampling to 250 Hz, epoch extraction, and baseline correction by removing the mean of each epoch. Epochs were extracted from −500 to 4,000 ms relative to the onset of the timed portion of each trial.

### 2.5 Feature extraction

The Filter Bank Common Spatial Patterns (FBCSP) algorithm was used to extract spatio-spectral features from the EEG signals (Ang et al., 2012). FBCSP has been widely applied in motor imagery BCI research and was previously shown to effectively capture features associated with singing imagery (Mohammadpour and Power, 2025). Because the spectral characteristics of the dual imagery tasks were not known a priori, a densely sampled filter bank spanning 0–96 Hz was used to capture discriminative information across a broad range of frequencies. Multiple filter bandwidths (4, 8, 12, and 16 Hz) were included to provide different spectral resolutions, while overlapping wider bands reduced the likelihood of informative frequency components occurring near band boundaries. Following pre-processing, the EEG signals were filtered using symmetric linear-phase FIR bandpass filters with bandwidths of 4, 8, 12, and 16 Hz over the range of 0–96 Hz. No overlap was used for the 4 Hz filters, while the remaining filters used 50% overlap, resulting in a total of 73 frequency bands. CSP feature extraction was implemented using the MNE-Python library. For each band, the Common Spatial Pattern (CSP) algorithm was used to optimize 10 CSP filters (five pairs), yielding a total of 730 features.

### 2.6 Task classification via EEG

The main objective is to evaluate the possibility of using DI tasks to increase the number of commands for active BCIs. The critical first step was to determine if it is possible to differentiate the DI tasks and each of the constituent single tasks. Thus the following 3-class scenarios were investigated: (1) R vs. SI vs. R_*SI*_, (2) L vs. SI vs. L_*SI*_, and (3) F vs. SI vs. F_*SI*_.

Next, incorporating the rest state into these problems, the following 4-class scenarios were investigated: (1) R vs. SI vs. R_*SI*_ vs. REST, (2) L vs. SI vs. L_*SI*_ vs. REST, and (3) F vs. SI vs. F_*SI*_ vs. REST.

Finally, the feasibility of increasing the number of commands beyond six was investigated through 7 and 8-class classification of the single and dual tasks. The 8-class scenario covered all the tasks performed in this experiment, and the 7-class combinations were obtained by removing the tasks, one at a time, from the 8-class combination.

A regularized LDA classifier implemented using the scikit-learn (Python) library was used for classification. Classifier performance was evaluated using five runs of 10-fold cross-validation. For each cross-validation fold, the CSP filters and regularized LDA classifier were trained using only the training data, and the resulting model was then evaluated on the held-out test data.

A regularized LDA classifier was selected as a well-established and interpretable baseline for EEG-based motor imagery classification. Given the relatively small sample size and high-dimensional feature space, simpler linear models are less prone to overfitting and allow for a clearer assessment of task separability. The primary aim of this study is to evaluate the discriminability of dual imagery tasks rather than to optimize classification performance; therefore, more complex models were not explored in this work but represent an important direction for future research.

### 2.7 Correlation between classification accuracy and self-rating data

For each participant, Pearson correlation coefficients were calculated between the subject's overall 8-class classification accuracy and the proportion of trials assigned to each self-rated performance category (i.e., strong, moderate, weak, and wrong).

## 3 Results

### 3.1 Participants' task preferences and difficulty ratings

**Figure 2:**
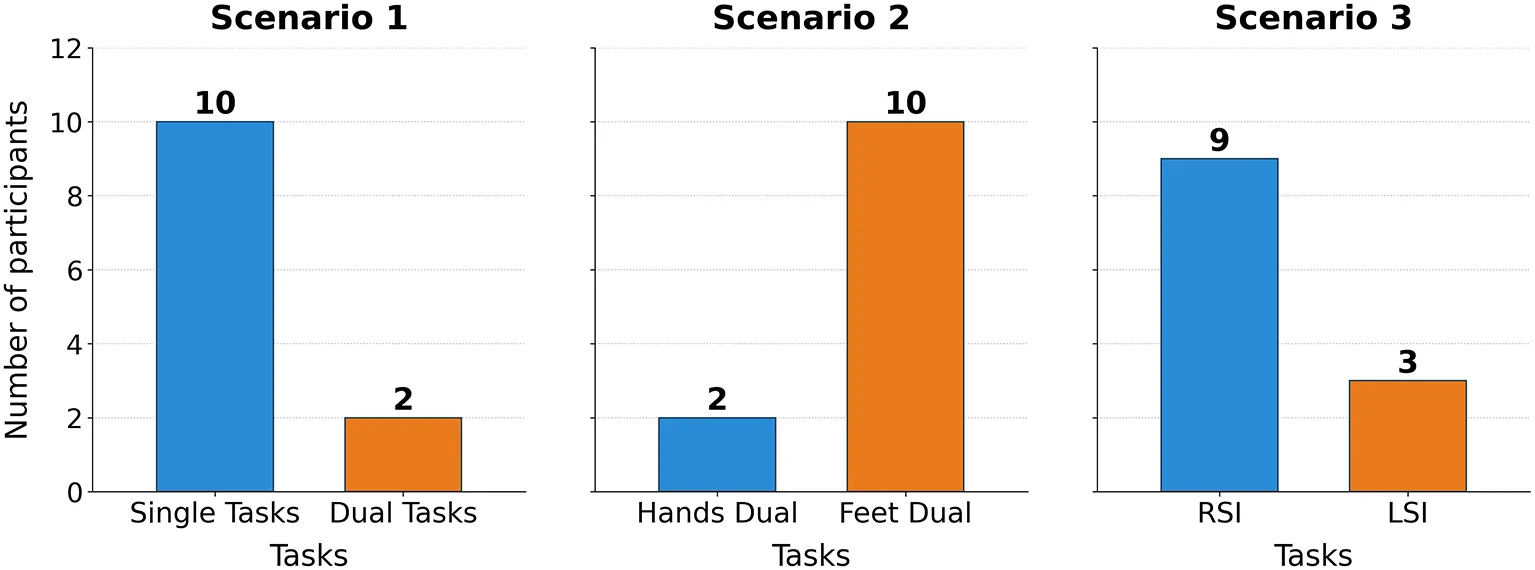
Participant responses to the task preference question.

Figure 2 illustrates the participants' responses to the question “*If you were to choose one of the tasks below to do on a daily basis to use a computer, which one would you pick?"*, when given the three different sets of options. For each scenario, the number of participants who preferred each option is indicated.

Also, participants rated the difficulty of each task on a five-point scale after completing Blocks 1, 4, and 6. The average difficulty ratings (across participants) are reported in Table 1. In this table, block average is the average perceived difficulty of all the tasks completed in that block, and task average is the average of the three perceived difficulty ratings taken for that specific task.

**Table 1:** Task difficulty ratings.

Task difficulty ratings				
	Recording after			
	Block 1	Block 4	Block 6	Task average
REST	1.2 (0.4)	1.3 (0.5)	1.3 (0.5)	1.2 (0.3)
R	2.2 (1.1)	2.1 (0.9)	1.8 (1.1)	2.0 (0.9)
L	2.3 (0.8)	2.2 (0.9)	1.9 (1.0)	2.1 (0.8)
F	1.9 (0.9)	2.0 (1.1)	1.8 (1.1)	1.9 (1.0)
SI	2.4 (1.2)	2.0 (0.9)	1.9 (0.7)	2.1 (0.9)
R_*SI*_	3.3 (1.4)	2.8 (1.0)	2.4 (1.0)	2.8 (1.0)
L_*SI*_	3.4 (1.1)	2.8 (0.9)	2.5 (0.9)	2.9 (0.9)
F_*SI*_	2.8 (1.3)	2.6 (1.2)	2.3 (1.1)	2.6 (1.2)
Block average	2.5 (1.0)	2.2 (0.9)	2.0 (0.9)	

### 3.2 EEG classification

**Figure 3:**
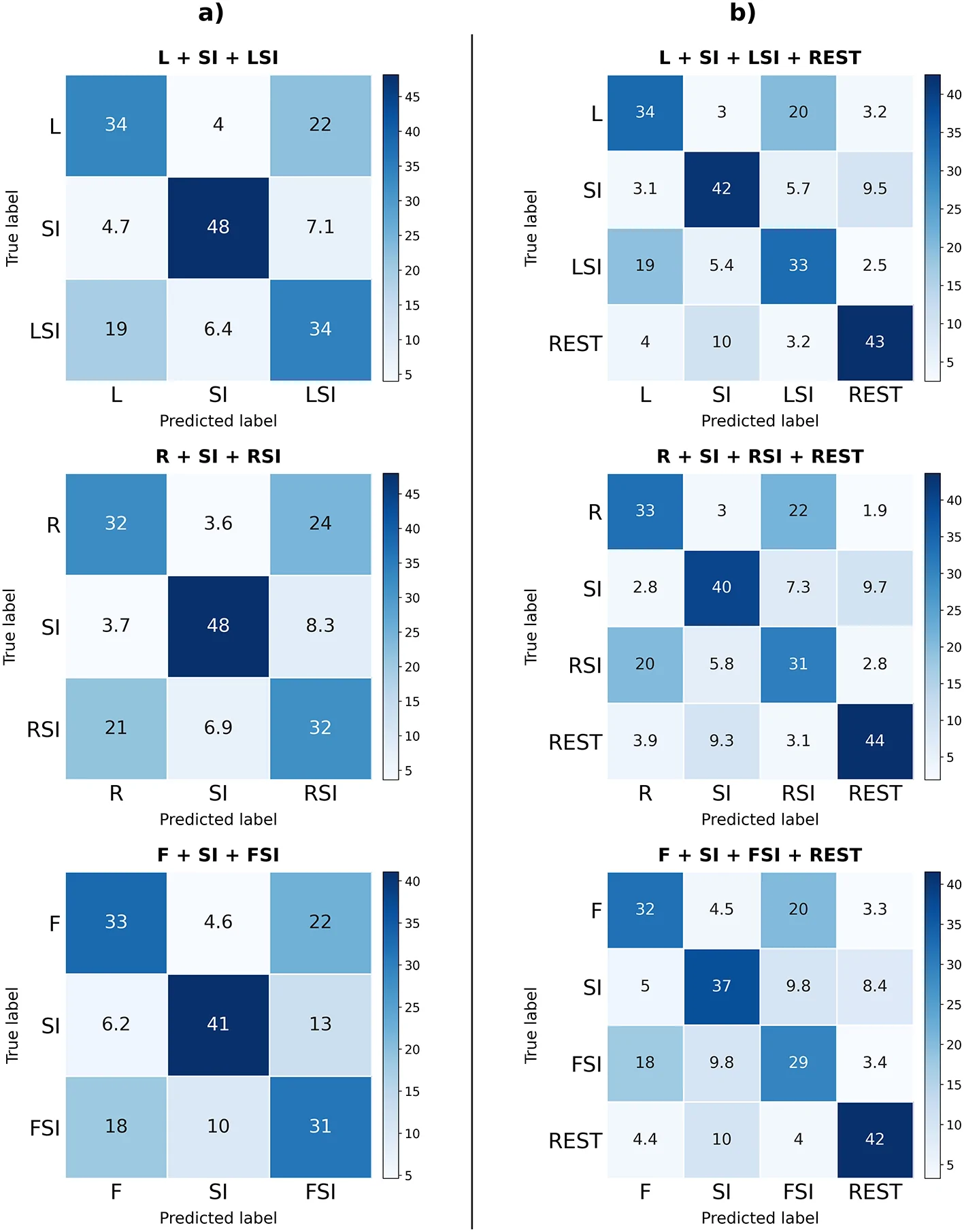
Confusion matrices for **(a)** 3-class scenarios, and **(b)** 4-class scenarios.

The results of the 3- and 4-class scenarios are shown in Table 2, including the overall accuracy, precision, recall and F1-score. Figure 3 shows the corresponding confusion matrices. All results are averaged across the 12 participants. For reference, Tables 2–4 also include participant-level binomial chance thresholds for the 3-, 4-, 7, and 8-class scenarios. These thresholds were calculated using the method described in Combrisson and Jerbi (2015) and based on the number of classified samples using a significance level of *p* = 0.05.

**Table 2:** Average classification results for the 3 and 4-class scenarios (%).

	3-Class scenarios			4-Class scenarios		
	L + SI + L_*SI*_			L + SI + L_*SI*_ + REST		
Overall accuracy	64.1 (13.9)			63.0 (14.9)		
Task	Precision	Recall	F1-score	Precision	Recall	F1-score
L	58.5 (14.4)	56.1 (14.0)	57.1 (14.1)	56.2 (14.4)	56.4 (15.5)	56.2 (15.0)
SI	82.8 (19.1)	80.3 (17.8)	81.4 (18.3)	69.4 (17.4)	69.8 (17.3)	69.5 (17.2)
L_*SI*_	53.6 (13.2)	57.0 (14.7)	55.1 (13.7)	53.6 (15.3)	55.0 (15.1)	54.2 (15.1)
REST	–	–	–	74.3 (16.4)	70.8 (15.5)	72.4 (15.8)
	**R + SI + R**_*SI*_			**R + SI + R**_*SI*_ **+ REST**		
**Overall accuracy**	**62.0 (11.3)**			**61.8 (12.6)**		
Task	Precision	Recall	F1-score	Precision	Recall	F1-score
R	55.8 (15.4)	53.4 (16.4)	54.4 (15.8)	55.6 (14.2)	56.1 (15.4)	55.7 (14.6)
SI	82.1 (14.7)	80.0 (15.1)	80.9 (14.8)	68.8 (14.8)	66.7 (15.9)	67.5 (15.1)
R_*SI*_	49.5 (10.2)	52.8 (10.2)	51.0 (10.0)	49.5 (10.6)	52.0 (11.3)	50.6 (10.9)
REST	–	–	–	75.5 (16.5)	72.5 (14.9)	73.9 (15.5)
	**F + SI + F**_*SI*_			**F + SI + F**_*SI*_ **+ REST**		
**Overall accuracy**	**59.3 (14.5)**			**58.1 (15.4)**		
Task	Precision	Recall	F1-score	Precision	Recall	F1-score
F	57.5 (11.6)	55.1 (11.3)	56.1 (11.3)	54.2 (14.0)	53.3 (15.5)	53.5 (14.4)
SI	73.5 (18.5)	68.4 (18.3)	70.7 (18.3)	60.7 (21.1)	61.3 (21.0)	60.9 (20.9)
F_*SI*_	47.5 (13.3)	52.4 (13.3)	49.7 (13.2)	46.7 (15.9)	48.7 (14.3)	47.6 (15.0)
REST	–	–	–	73.8 (17.1)	69.2 (16.4)	71.3 (16.6)

Note that the participant-level binomial chance thresholds for these scenarios are 38.9% and 29.6%, respectively.

The results of the 7-class combinations—which resulted from each task being removed, one at a time, from the full set of eight tasks—are reported in Table 3. The reported value is the average accuracy when the specified task was excluded from the classification.

**Table 3:** Average classification results for the 7-class scenarios.

7-Class scenarios (Participant-level binomial chance threshold: 17.1%)								
	MI				DI			
	L	R	F	SI	L_*SI*_	R_*SI*_	F_*SI*_	REST
Accuracy	53.8 (16.7)	52.8 (16.7)	52.2 (15.1)	51.6 (16.4)	54.2 (16.4)	55.4 (16.1)	55.1 (15.3)	49.9 (15.3)

**Figure 4:**
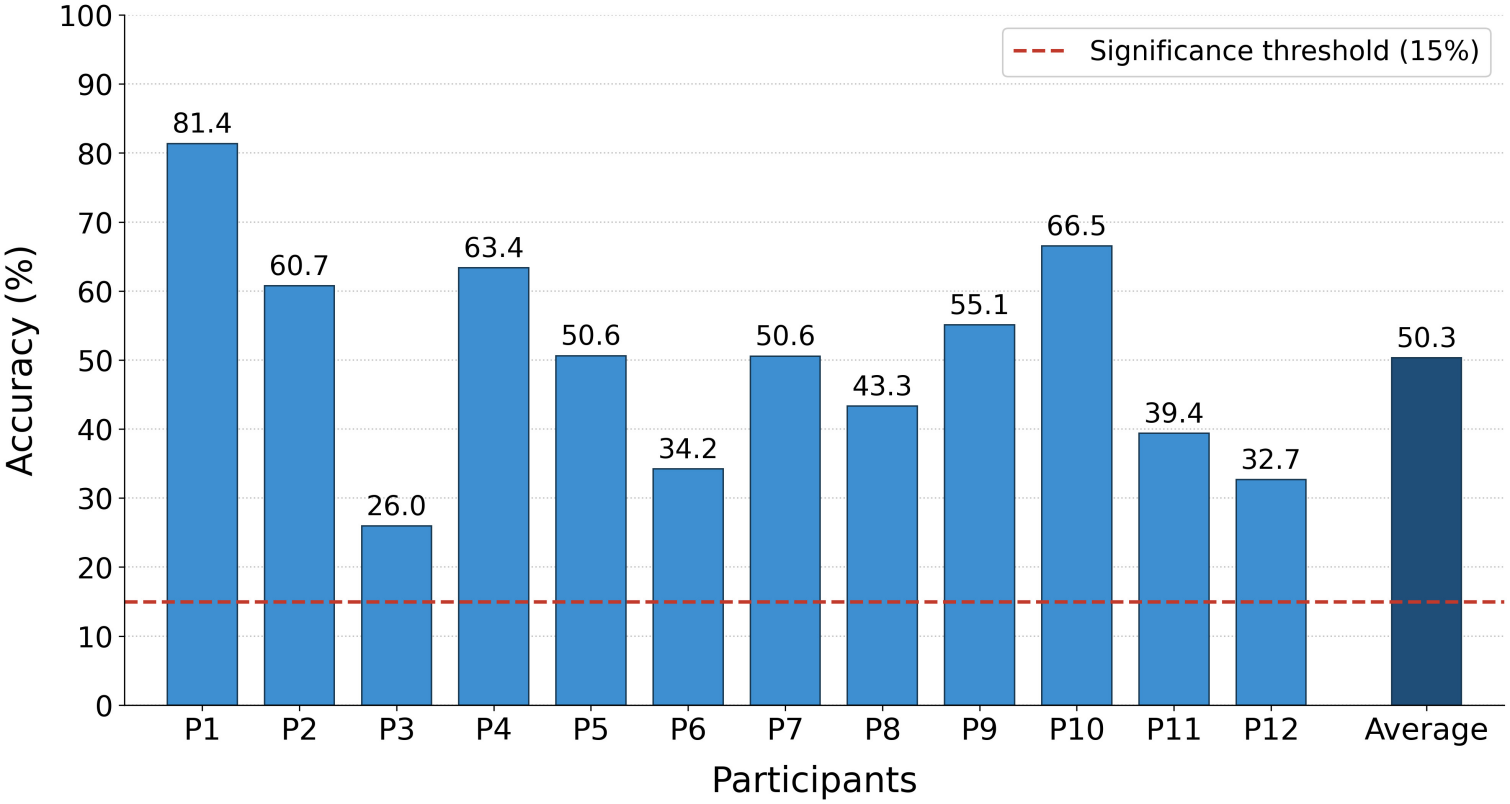
Individual participant accuracies for the 8-class scenario.

For the 8-class scenario, which includes all of the tasks considered in this study, an average accuracy of 50.3 ± 16.1% was achieved. Table 4 shows the average performance evaluation metrics (precision, recall, and F1-score) for each of the eight tasks. The individual participant accuracies for the 8-class scenario are shown in Figure 4.

**Table 4:** Average classification metrics for the 8-class scenario (%).

8-class scenario								
	MI				DI			
Metric	L	R	F	SI	L_*SI*_	R_*SI*_	F_*SI*_	REST
Precision	49.3 (17.7)	52.3 (16.7)	48.2 (17.6)	52.2 (19.3)	45.1 (19.0)	43.4 (16.2)	41.4 (18.5)	69.0 (18.4)
Recall	50.3 (18.3)	51.9 (14.8)	50.0 (19.6)	49.3 (18.5)	46.2 (21.1)	44.0 (17.3)	41.3 (16.1)	64.8 (18.4)
F1-score	49.7 (17.9)	52.0 (15.6)	49.0 (18.5)	50.6 (18.8)	45.5 (19.9)	43.6 (16.6)	41.3 (17.3)	66.6 (18.1)

**Figure 5:**
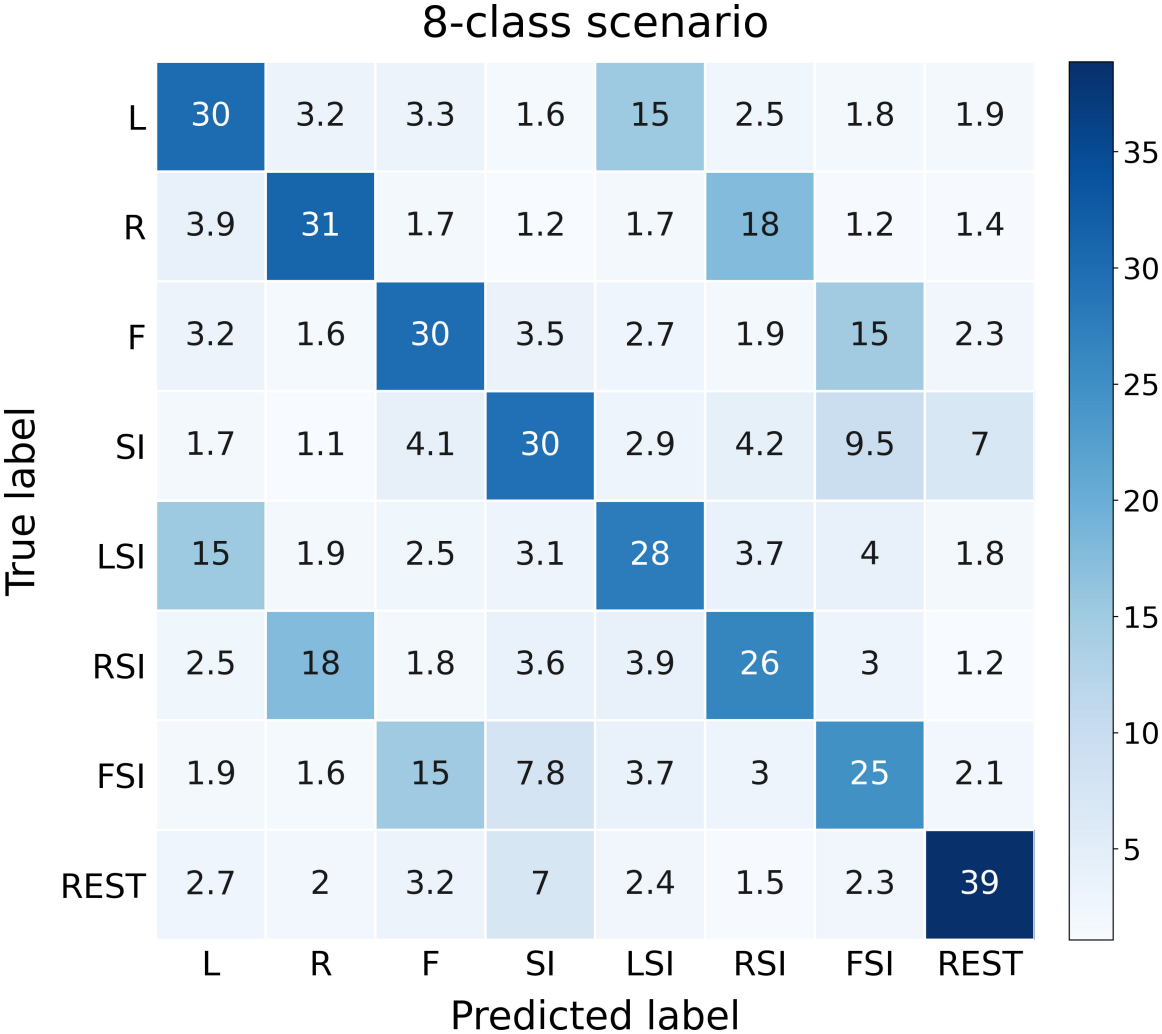
Confusion matrix for the 8-class scenario.

Figure 5 shows the average confusion matrix for the 8-class scenario and demonstrates the common classifier errors.

### 3.3 Correlation between classification accuracy and self-rating data

**Figure 6:**
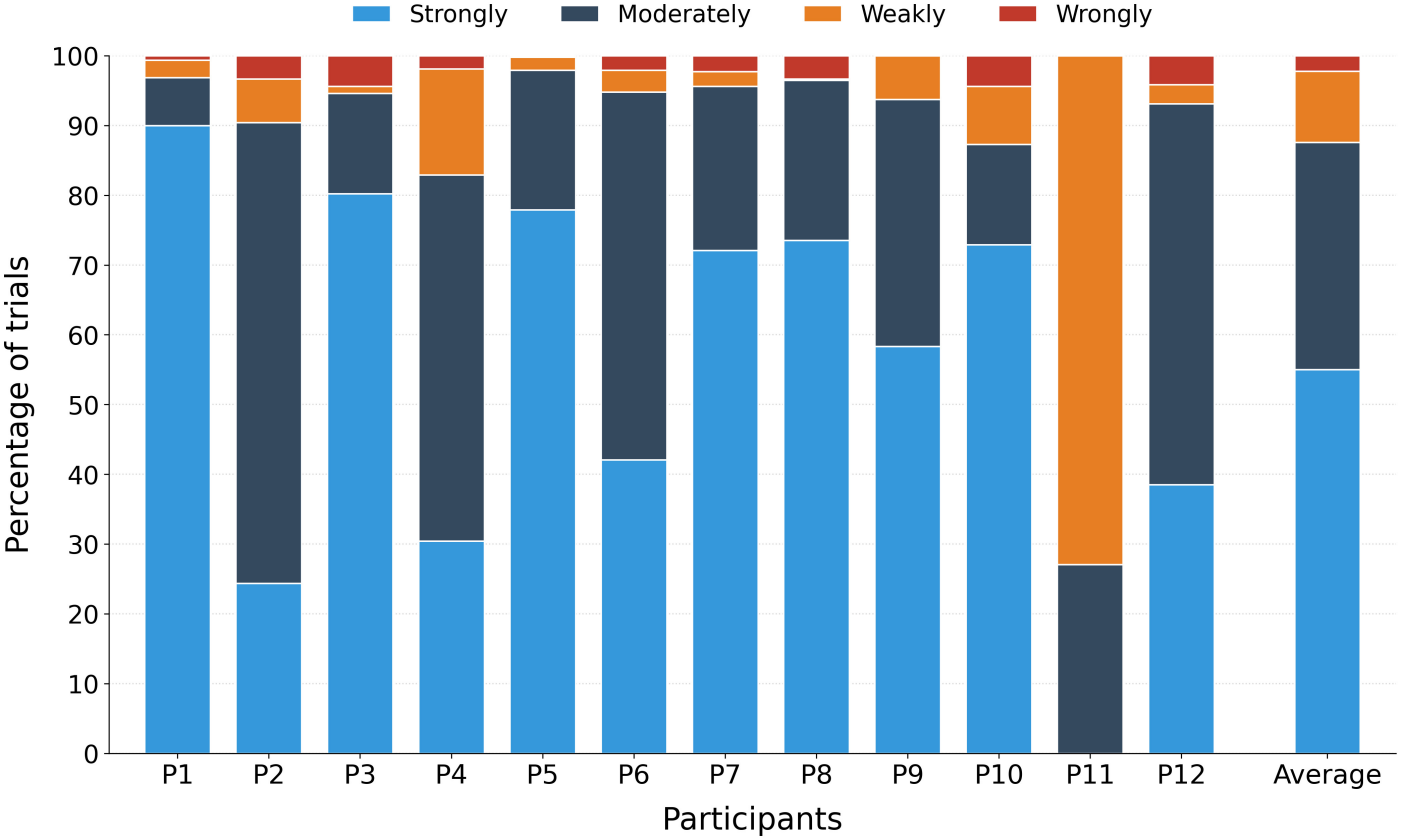
Results of self-rating for all participants.

Figure 6 is a summary of the results of self-rating for each participant, reporting the percentage of trials for which they indicated their performance was “strong," “moderate," “weak," and “wrong."

None of the performance ratings demonstrated a significant correlation with the 8-class classification accuracy (Pearson correlations; all *p*>0.23).

## 4 Discussion

### 4.1 Task preferences and difficulty ratings

Target users for active BCIs are typically individuals with severe physical disabilities. These systems have the potential to provide such individuals with a movement-free means of environmental control and allow the users to communicate their desired commands with just the imagination of different tasks. The difficulty and intuitiveness of these tasks can significantly impact their overall experience with the device and, as a result, their quality of life. Thus, any new control task that is considered for use in an active BCI needs to be suitable for long-term use.

In this experiment, we asked the participants to perform dual imagery tasks, which are clearly more difficult than single tasks. It is not unexpected that 10 out of 12 participants found MI tasks preferable to DI tasks. Also, it was quite expected for participants to rate DI tasks as more difficult than MI and SI.

Encouragingly, however, a downward trend in participant difficulty ratings across the session was observed for all tasks (except REST), with the decrease in difficulty rating being larger for the DI tasks than for the single tasks. Specifically, while the average difficulty rating for the single tasks reduced by about 0.3, from around 2.2 after block 1 to about 1.9 by the end of the session, the average DI task difficulty ratings changed by about 0.8, from about 3.2 to 2.4 over the same number of trials. Therefore, it appears that the initial difficulty of performing DI tasks was considerably reduced as participants spent more time performing the tasks and gained more familiarity with them, and the difficulty ratings of DI ended up being markedly closer to those of the single MI and SI tasks. Presumably, with additional practice/training, the perceived difficulty of performing the DI tasks could reduce even further.

All of the participants in this study were right-handed, and nine out of 12 individuals preferred performing DI with their dominant hand (i.e., right) vs. their non-dominant hand. Moreover, the difficulty ratings appear to corroborate the responses to the task preference questions, which indicated that participants generally preferred the F_*SI*_ task to the R_*SI*_ and L_*SI*_ tasks—F_*SI*_ had a lower average difficulty rating (2.6/5) in comparison to the other two tasks (2.8/5 and 2.9/5, respectively). Overall, it seems that participants found F_*SI*_ to be a relatively more intuitive option.

### 4.2 EEG classification results

#### 4.2.1 Dual vs. Single task classification

The first concern in using DI tasks for increasing the number of commands is the feasibility of differentiating the DI tasks and each of the constituent single tasks (e.g., L vs. SI vs. L_*SI*_). The results of the 3- and 4-class scenarios demonstrate that a MI task, SI, and their corresponding dual task are differentiable via EEG with accuracies well above chance. This was true for all task combinations that were investigated. In all cases, the precision and recall metrics of the individual tasks were also well above chance.

The results in Figure 5 also provide insight into the observed classification errors. The greatest mutual confusion occurred between each dual imagery task and its corresponding motor imagery task (L-L_*SI*_: 15% in each direction, R–R_*SI*_: 18% in each direction, and F-F_*SI*_: 15% in each direction). In contrast, confusion between SI and the corresponding dual imagery tasks was substantially lower (SI-L_*SI*_: 2.9%/3.1%, SI-R_*SI*_: 4.2%/3.6%, and SI-F_*SI*_: 9.5%/7.8%). Thus, the dual imagery tasks were more frequently confused with their corresponding motor imagery tasks than with singing imagery alone. The F_*SI*_ task exhibited greater confusion with SI than did R_*SI*_ and L_*SI*_, which is consistent with the slightly lower classification accuracies obtained for the 3- and 4-class combinations based on F, SI, and F_*SI*_.

The observed confusion pattern is consistent with greater overlap between the dual imagery tasks and their corresponding motor imagery tasks than between the dual imagery tasks and singing imagery alone. However, alternative explanations, including differences in participants' execution of the dual imagery tasks, may also have contributed to the observed confusion.

One possible contributor to the observed confusion patterns was suggested by the informal feedback received from participants, which indicated that some individuals found it difficult to “turn on" the imagination of singing the song for the DI tasks and then “turn it off" for the single tasks. This could have negatively impacted the classification results by making the different imagery tasks more difficult to distinguish. With training and practice, this could become easier for participants. However, it should also be noted that controlling a BCI using a mental activity that people very commonly perform (which is certainly true of singing imagery for most people) may result in false positives if the user “accidentally" performs the task without the intention of sending a command.

Interestingly, the direct confusion between the dual imagery tasks and their corresponding motor imagery tasks was lower in the 8-class scenario than in the 3- and 4-class scenarios, despite the increase in the number of classes.

Incorporating REST, or a “no control" state, into an active BCI can significantly enhance the practicality of the device. Having the BCI be able to recognize an idle state means that the user would not be required to perform a specific task during every control period (they could, for example, just do nothing if they do not wish to send a command). Consistent with this objective, the REST condition was intentionally left unconstrained, apart from requiring participants to keep their eyes open and focused on the screen and refrain from performing any of the mental imagery tasks, so as to better reflect a practical BCI scenario in which the user is not intentionally generating a control command. The 4-class analyses suggest that all of the single tasks and their dual versions are distinguishable from REST with accuracies well above chance, with the MI and DI tasks appearing to be more differentiable from REST than is SI (i.e., the SI task is more often mislabelled as REST than are MI and DI). Although a more constrained resting condition (e.g., requiring visual fixation or another controlled baseline task) could potentially improve discrimination from mental imagery tasks, this would represent a trade-off between classification performance and the ecological validity of the intended BCI application.

Similarly, participants were asked to imagine different familiar songs across experimental blocks rather than repeatedly imagining a single predetermined song. This design was intended to better reflect a practical BCI scenario, in which users would naturally imagine different songs over time, and to encourage the classifier to learn neural patterns associated with the cognitive process of singing imagery rather than relying on neural activity associated with repeatedly imagining a single song. Nevertheless, differences in song characteristics, such as rhythm or emotional associations, may have introduced additional variability into the EEG signals. Future studies could investigate the influence of these factors on classification performance.

#### 4.2.2 Increasing the number of commands

In our previous study (Mohammadpour and Power, 2025), the potential of designing a 6-class active BCI that incorporated the SI task into MI-BCI was investigated, and accuracies as high as 60.7 % were achieved. Here, we explored the possibility of increasing the number of commands beyond that number. Specifically, 7- and 8-class scenarios were explored to evaluate the potential utility of DI tasks in multi-class active BCI. The 8-class scenario yielded a very encouraging average accuracy of 50.3%, substantially higher than the theoretical chance level of 12.5%.

In addition, all possible 7-class combinations (obtained by excluding one of the eight tasks in each case) were considered. The highest 7-class accuracy yielded a 5.1 percentage-point improvement as compared to the 8-class scenario, at the expense of one less command. This accuracy was obtained by excluding the R_*SI*_ task, which was not surprising given that the confusion matrix for the 8-class scenario indicates that the most common error was misclassifying R as R_*SI*_ or vice versa. Also, R_*SI*_ and F_*SI*_ were most frequently misclassified. Consequently, 7-class scenarios in which one of these two tasks was removed led to higher classification accuracies.

While the results obtained here are very promising and show the potential of using DI tasks in active BCI, the accuracies obtained for all of the 7- and 8-class combinations explored were below the 70% threshold, which is often said to be required for efficient BCI communication (Kübler et al., 2001). Nevertheless, it may be reasonable to assume that as participants become more familiar and comfortable with the DI tasks, classification accuracy could be enhanced. It should be noted that the present study was conducted within a single experimental session with limited participant training; prior work in motor imagery BCIs has shown that performance can improve substantially with extended practice and user-specific adaptation. As such, the accuracies reported here may underestimate the achievable performance of dual imagery paradigms under more extensive training conditions. Furthermore, as this was a feasibility study involving a relatively small sample of right-handed participants, the findings should be considered preliminary. Future studies involving larger and more diverse cohorts, including left-handed participants, will be important for establishing the generalizability of these results. Additionally, improvements in feature extraction, selection, or classification algorithms could further enhance performance. In particular, advanced approaches such as deep learning and Riemannian geometry-based classifiers have shown promise in EEG classification and represent important directions for future investigation. However, the extent of any improvement for the proposed dual imagery paradigm remains to be established.

### 4.3 Self-rating data

The observed lack of correlation between participants' self-assessments of how well they performed the imagery tasks and their 8-class classification accuracies suggests that perceived success in task execution does not necessarily correspond to the distinctiveness of the resulting EEG patterns. Participants may have accurately judged that they performed the instructed imagery as intended while still producing neural activity that was difficult for the classifier to distinguish from other imagery tasks. Consequently, self-assessed task performance should not be interpreted as a direct indicator of expected classification accuracy.

## 5 Conclusion

The results of this study indicate that incorporating DI tasks may be a feasible approach for increasing the number of distinguishable control states in active BCIs. Specifically, an average accuracy of approximately 50% was achieved for the 8-class scenario, substantially above the theoretical chance level of 12.5%. While the obtained accuracies are currently insufficient for practical high-command BCI systems, the results demonstrate the potential of combinatorial imagery paradigms for expanding the set of distinguishable mental states available for EEG-based active BCIs.

## Data Availability

The datasets presented in this article are not readily available because participants did not consent to having their data shared beyond the research team. Requests to access the datasets should be directed to Sarah D. Power, sd.power@mun.ca.
